# Methotrexate monotherapy for unilateral moderately active thyroid‐related eye disease

**DOI:** 10.1002/ccr3.4559

**Published:** 2021-07-09

**Authors:** Ayman G. Elnahry, Joseph H. Talbet, Gehad A. Elnahry

**Affiliations:** ^1^ Department of Ophthalmology Faculty of Medicine Cairo University Cairo Egypt; ^2^ College of Medicine Howard University Washington DC USA

**Keywords:** euthyroidism, methotrexate, monotherapy, thyroid‐related eye disease

## Abstract

A type 1 diabetic patient with unilateral active thyroid‐related eye disease was intolerant to systemic steroid therapy due to uncontrollable blood sugar levels. She was treated with low‐dose methotrexate monotherapy, which resulted in a marked improvement of her condition with no adverse events.

## Graphical abstract

Active thyroid‐related eye disease may be safely and effectively managed using low‐dose methotrexate monotherapy especially in patients intolerant to steroid therapy.

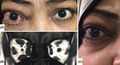

## 1 CASE DESCRIPTION

**FIGURE 1:**
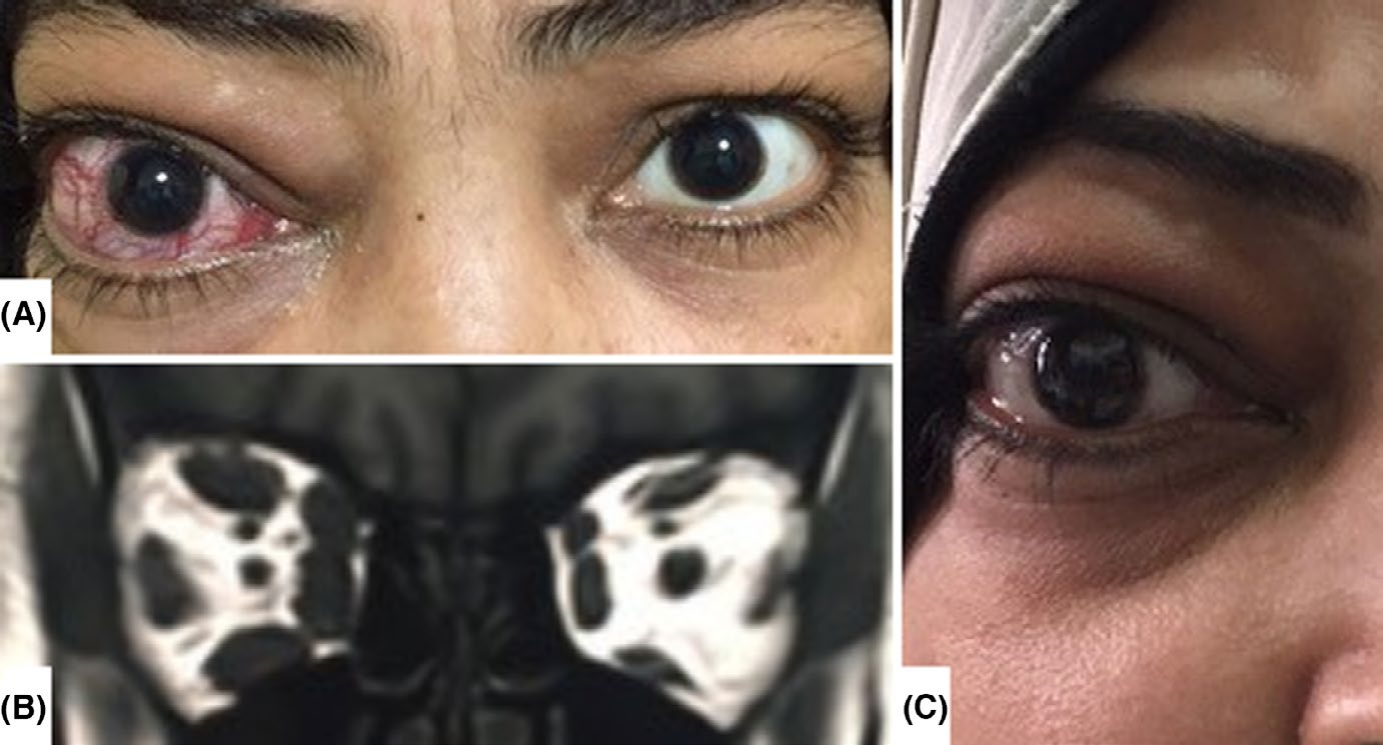
At presentation, there was redness, proptosis, upper eyelid edema and retraction, and increased inferior scleral show of the right eye compared with the left, with a clinical activity score (CAS) of 5/7 (A). Magnetic resonance imaging of the orbit revealed an enlargement of the extraocular muscles of the right eye with tendon sparing compared with the left (B). Six months following methotrexate monotherapy, there was marked improvement of the clinical condition apart from mild residual conjunctival chemosis (C)

A 43‐year‐old diabetic woman presented with a red painful right eye (RE) for 1 month. She had normal vision with no afferent pupillary defect. The RE had elevation and abduction limitation, lid edema, proptosis (RE: 23 mm protrusion, left eye: 20 mm), and Dalrymple's sign (Figure 1A), with dilated RE conjunctival vessels and normal anterior and posterior segments. MRI revealed right extraocular muscle enlargement (Figure 1B). Liver functions, free‐T3, free‐T4, and thyroid‐stimulating hormone (TSH) were unremarkable. However, TSH‐receptor antibodies were positive. She was diagnosed with unilateral active thyroid‐related eye disease (TED) and prescribed 60 mg/day oral prednisone with lubricating eye drops. After 2 weeks, however, she was steroid intolerant due to uncontrollable blood sugar and was switched to 10 mg methotrexate weekly. Her condition gradually improved, and six months later, her CAS was 2/10 (Figure 1C). Methotrexate was gradually tapered over 3 months without relapse for 9 months. During treatment, laboratory investigations remained normal.

TED usually occurs with hyperthyroidism but can also occur with hypothyroidism/euthyroidism.^1^ It can be unilateral or bilateral and requires detecting anti‐thyroid antibodies for diagnosis in euthyroidism. The mainstay of treating moderate‐severe TED is glucocorticoids; however, methotrexate monotherapy can be a safe and effective alternative by reducing cellular proliferation.^2^

## CONFLICT OF INTEREST

None.

## AUTHOR CONTRIBUTIONS

AGE: has made substantial contributions in the acquisition and interpretation of data, and in drafting of the manuscript. JHT: has made substantial contributions in the analysis and interpretation of the data, and in revising the manuscript. GAE: treated the patient and has made substantial contributions in the acquisition and interpretation of data, and in revising the manuscript. All authors read and approved the final version of the manuscript and agree to be accountable for all aspects of the work.

## ETHICAL STATEMENT

Written informed consent for publication of photographs was obtained from the patient and is available upon request. This case report did not receive any funding. Authors have access to all source data for this case report. This report was approved by Cairo University research ethics committee and followed the tenets of the Declaration of Helsinki.

## Data Availability

All the data used in this report are available within the article.
